# Characterization, antimicrobial and antitumor activity of superoxide dismutase extracted from Egyptian honeybee venom (*Apis mellifera lamarckii*)

**DOI:** 10.1186/s43141-023-00470-4

**Published:** 2023-02-20

**Authors:** Mohamed M. Abdel-Monsef, Doaa A. Darwish, Hind A. Zidan, Ahmed A. Hamed, Mahmoud A. Ibrahim

**Affiliations:** 1 Molecular Biology Department National Research Centre, Protium Research Laboratory, Dokki, Giza, Egypt; 2 Agricultural Research Center, Plant Protection Research Institute, Giza, Egypt; 3 Microbial Chemistry Department, National Research Centre, Dokki, Giza, Egypt

**Keywords:** Bee venom, Superoxide dismutase, Purification and characterization, Antimicrobial, Antitumor

## Abstract

**Background:**

Superoxide dismutase is an important antioxidative stress enzyme which is found in honeybee venom and has a wide pharmaceutical and medical applications.

**Results:**

We reported the purification and characterization of venom SOD from Egyptian honeybee *Apis mellifera lamarckii* and termed BVSOD. It was purified to homogeneity from the Egyptian honeybee venom. The purification procedures included crude extraction, DEAE-cellulose anion exchange column chromatography, and Sephacryl S-300 gel filtration column chromatography. The purified BVSOD is found to be homogeneous as investigated by native PAGE. It exhibited homodimeric structure with a molecular weight of native form of 32 kDa and subunits of 16.0 kDa. It displayed the maximum activity at pH 7.4. CuCl_2_, ZnCl_2_, and MgCl_2_ and elevated the activity of BVSOD, while CoCl_2_, FeCl_2_, and NiCl_2_ inhibited BVSOD activity. Potassium cyanide and hydrogen peroxide were most potent inhibitors for BVSOD activity suggesting that it is a Cu/Zn-SOD type.

**Conclusions:**

The purified BVSOD is found to have antimicrobial and antitumor activities which can be used for various medical and clinical applications.

## Background

Superoxide dismutases (SODs) are a family of antioxidant metalloenzymes which defend against oxidative stress resulting from reactive oxygen species by scavenging superoxide radicals [1, 2]. SODs are catalyzing the disproportionation of superoxide free radicals to H_2_O_2_ and H_2_O to protect cells against their harmful effects [3, 4]. SODs are classified into four groups according to active site metal species, Mn-SOD, Cu/Zn-SOD, Ni-SOD, and Fe-SOD [5–9]. SODs have increasing commercial applications in cosmetics, clinical nutrition, and pharmaceutical industries [10]. SODs are used as antioxidant drugs in many diseases [11]. SODs are found to have antiviral and anti-inflammatory activities [12]. SODs have been studied in many of insect species, and it plays a protective role against oxidative stress caused by environmental stressors such as cold, heat, heavy metals, starvation, insecticides, and pathogens [13–16]. Bee venom is a pharmacologically complex mixture containing active enzymes, peptides, and proteins [17]. Bee venom is a secretion of bee’s sting apparatus and used to protect bees from any enemies [18, 19]. Bee venom is found to have antibacterial, anti-inflammatory, radioprotective, antimutagenic, immunity-promoting, antinociceptive, anticancer, and hepatocyte-protective activities so it is used in treating many diseases in folk medicine [20–24]. The aim of the present work is to purify SOD from the Egyptian honeybee venom *Apis mellifera lamarckii*, comprehensively discuss their characteristics, with emphasis on their potential antibacterial and antitumor activities.

## Materials and methods

### Materials

#### Collection of venom

The honeybee colonies were obtained from governorate of Asuit Egyptian subspecies *Apis mellifera lamarckii*. Venom was extracted from workers. Five-hundred workers of foraging bees were catched at the colony entry and rapid freezed at − 20 °C to immobilized. Bees were handly dissected; venom reservoir and sting device were removed, then disrupted in Eppendorf with 2.5 ml of H_2_O, and then centrifuged at 12,000 × g, 5 min, 4 °C. The supernatants were obtained and designated venom extract.

#### Chemicals

Xanthine sodium salt, xanthine oxidase enzyme, cytochrome C from horse heart, nitroblue tetrazolium (NBT), dimethyl sulfoxide (DMSO), phenyl methosulfate (PMS), phenymethylsulfonyl fluoride (PMSF), 1,4 dithiothreitol (DTT), 1,10 phenanthroline, trypan blue dye, bovine serum albumin (BSA), blue dextran, crystal violet, Sephacryl S-300, DEAE cellulose, and kits of gel filtration molecular weight marker were product of Sigma Co. SDS molecular weight marker proteins were purchased from Pharmacia Co., DMEM, fetal bovine serum, HEPES buffer solution, RPMI-1640, gentamycin, and L-glutamine are purchased from Lonza, Belgium.

## Methods

### SOD activity assay

SOD activity assay was based on the SOD ability to inhibit reduction of cytochrome C by scavenging superoxide anion formed by xanthine-xanthine oxidase system. The reaction mixture assay is 1.0 ml buffer 20 mmol L^−1^ potassium phosphate (pH 7.8), containing 0.1 mM EDTA, 0.01 mM cytochrome C, and 0.05 mM sodium xanthine. Reaction started by 21 munit of xanthine oxidase that reacts with sodium xanthine (substrate) producing superoxide anion that makes a reduction to cytochrome C at 550 nm. One unit activity of SOD is the amount which causes 50% reaction inhibition for reduction rate of cytochrome C [25].

### Staining of SOD activity on polyacrylamide gels

Staining of SOD activity was determined according to Weisiger and Fridovich method [26]. Determination of activity of SOD following electrophoresis was carried out by a reaction mixture containing phenazine methosulfate (PMS) and nitroblue tetrazolium salt (NBT) that produced superoxide anions, and then, formazan was formed by reduction of NBT by superoxide. Achromatic zones appeared on gel where superoxide radicals disappeared due to SOD activity preventing the NBT reduction. A buffered reaction mixture of NBT and PMS applied on gels and then exposed for minutes to sun daylight until achromatic zones indicating SOD activity formed on a blue background of gel.

### Purification of bee venom superoxide dismutase

The BVSOD was isolated from *Apis mellifera lamarckii* by two chromatographic steps: chromatography on DEAE-cellulose column and Sephacryl S-300 column. Venom extract was loaded onto DEAE-cellulose column (12 cm × 2.4 cm i.d.) pre-equilibrated with 20 mmol L^−1^ potassium phosphate buffer (pH 7.4). The elution of adsorbed proteins was carried out with stepwise NaCl gradient of 0 to 1 M in the same buffer at flow rate of 60 ml/h. Fractions of 3 ml were collected and analyzed for protein content and enzyme activity. Fractions containing SOD activity were pooled and concentrated. The concentrated enzyme was processed on a column Sephacryl S-300 (142 cm × 1.75 cm i.d.) which equilibrated and run with 20 mmol L^−1^ potassium phosphate buffer (pH 7.4) in a 30 ml/h flow rate. Fractions of 2 ml volume containing SOD were pooled. The protein concentration was measured following the method of Bradford, by using bovine serum albumin as standard [27].

### Native and SDS/PAGE gel electrophoresis

Purification steps, venom extract, chromatography on DEAE-cellulose column, and chromatography on Sephacryl S-300 column fraction are investigated by 7% native PAGE electrophoresis. Gel electrophoresis was achieved using 7% native PAGE using method of Smith [28] and SDS/PAGE which was carried out with 12% PAGE using method of Laemmli [29]. The molecular weight of purified SOD enzyme was determined using SDS-PAGE according to Weber and Osborn [30]. Proteins stained using Coomassie brilliant blue (R-250) 0.25% conc. SOD activity stained on PAGE according to Weisiger and Fridovich [26].

### Antimicrobial activity determination

A total of 500 µg/ml of purified BVSOD was dissolved by methanol, and 50-μl aliquots were soaked on discs of filter paper (Whatman No. 1) and dried [31]. The discs were distributed on agar plates surface inoculated with microbes to be tested and incubated for 24 h at 37 °C for bacteria and 48 h at 30 °C for fungi and yeast. Yeast and bacteria were grown on nutrient agar; fungi were grown on PDA (DSMZ 130) medium. After incubation, the inhibition zones of diameter were measured for tested microorganisms: *Proteus mirabilis* ATCC 25,933, *Salmonella typhi* ATCC6539, *Klebsiella pneumoniae* ATCC 43,816, *Candida albicans* ATCC10231, and *Aspergillus niger* NRRLA-326. Oxytetracycline (OT) was used as reference standard. The tested microbes were obtained from National Research Center, Egypt, Microbial Chemistry Department, Culture Collection Center.

### Antitumor activity determination

Human hepatocellular carcinoma cell line (HepG-2 cells) and human breast cancer cell line (MCF-7 cells) were obtained from VACSERA, Egypt, Tissue Culture Unit. The cells were propagated in medium of Dulbecco’s modified Eagle with HEPES buffer, 50 µg/ml gentamycin, 10% heat-inactivated fetal bovine serum, and 1% L-glutamine. Cells were incubated in 5% CO_2_ at 37 °C and subcultured two times a week. For assay of cytotoxicity, 100 µl medium was seeded in 96-well plate with 1 × 10^4^ cells/well concentration. Fresh growth medium containing purified BVSOD with different concentrations was added. After incubation for 24 h at 37 °C, the yield of viable cells was spectrophotometrically determined at 490 nm. Treated cells and control cells (in the absence of purified BVSOD) were compared. The cytotoxic effect of purified BVSOD was calculated by [(ODt/ODc)] × 100% where ODt is optical density mean of treated wells with purified BVSOD, and ODc is optical density mean of untreated cells. The survival curve (relation between purified BVSOD concentration and surviving cells) was plotted. A total of 50% concentration of inhibition (*IC*_50_) was determined from graphic plots of curve for each concentration. The microscopic examination of the tumor cells treated with purified BVSOD and control cells was carried out. The cells were stained, and cellular morphology was investigated using an inverted microscope and a digital microscopy camera to capture the images [32–34].

## Results

### Purification of superoxide dismutase

**Fig. 1:**
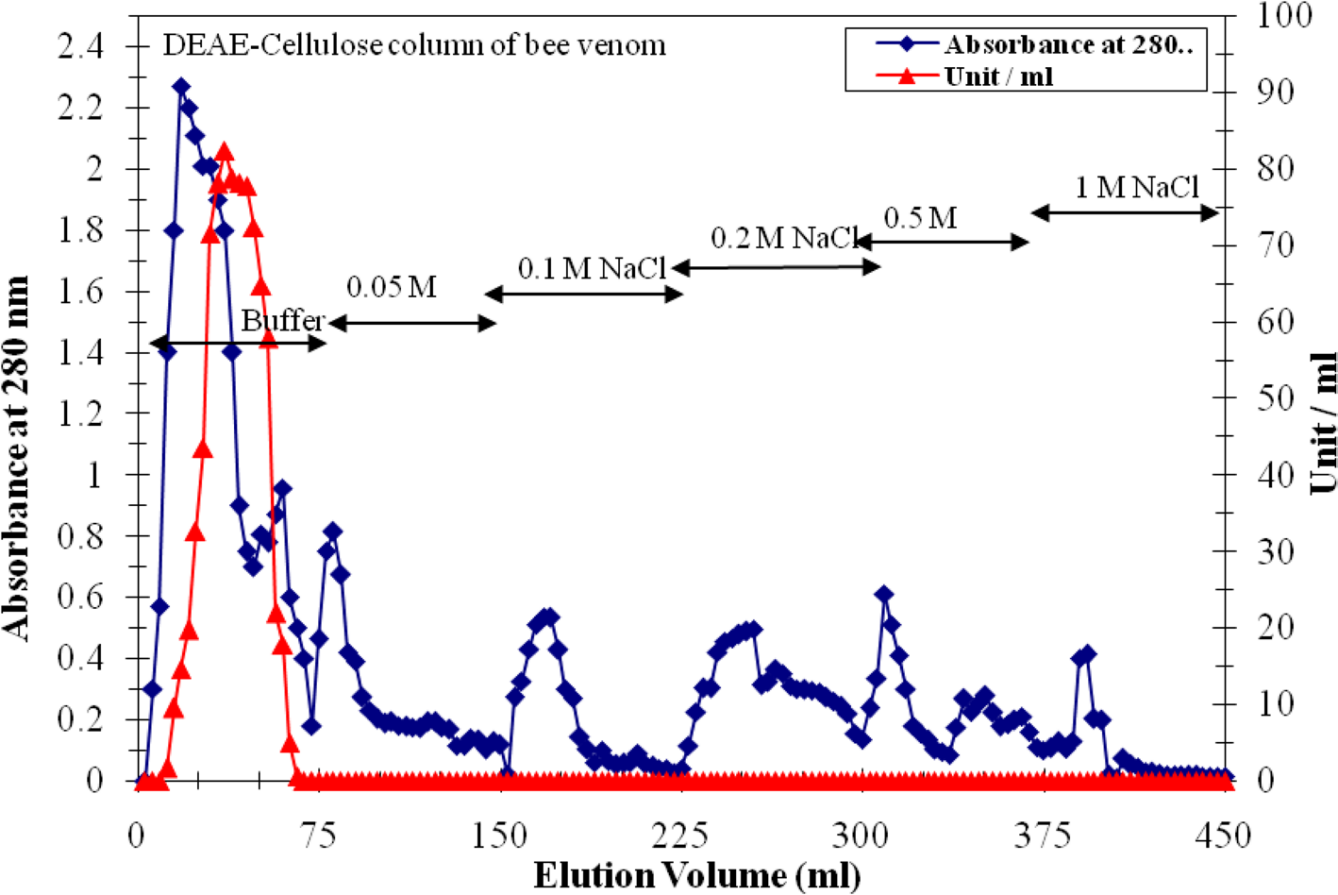
A chromatographic typical elution profile for honeybee venom extract on DEAE-cellulose column (12 cm × 2.4 cm i.d.) previously equilibrated with 20 mmol L.^−1^ potassium phosphate buffer (pH 7.4)

**Fig. 2:**
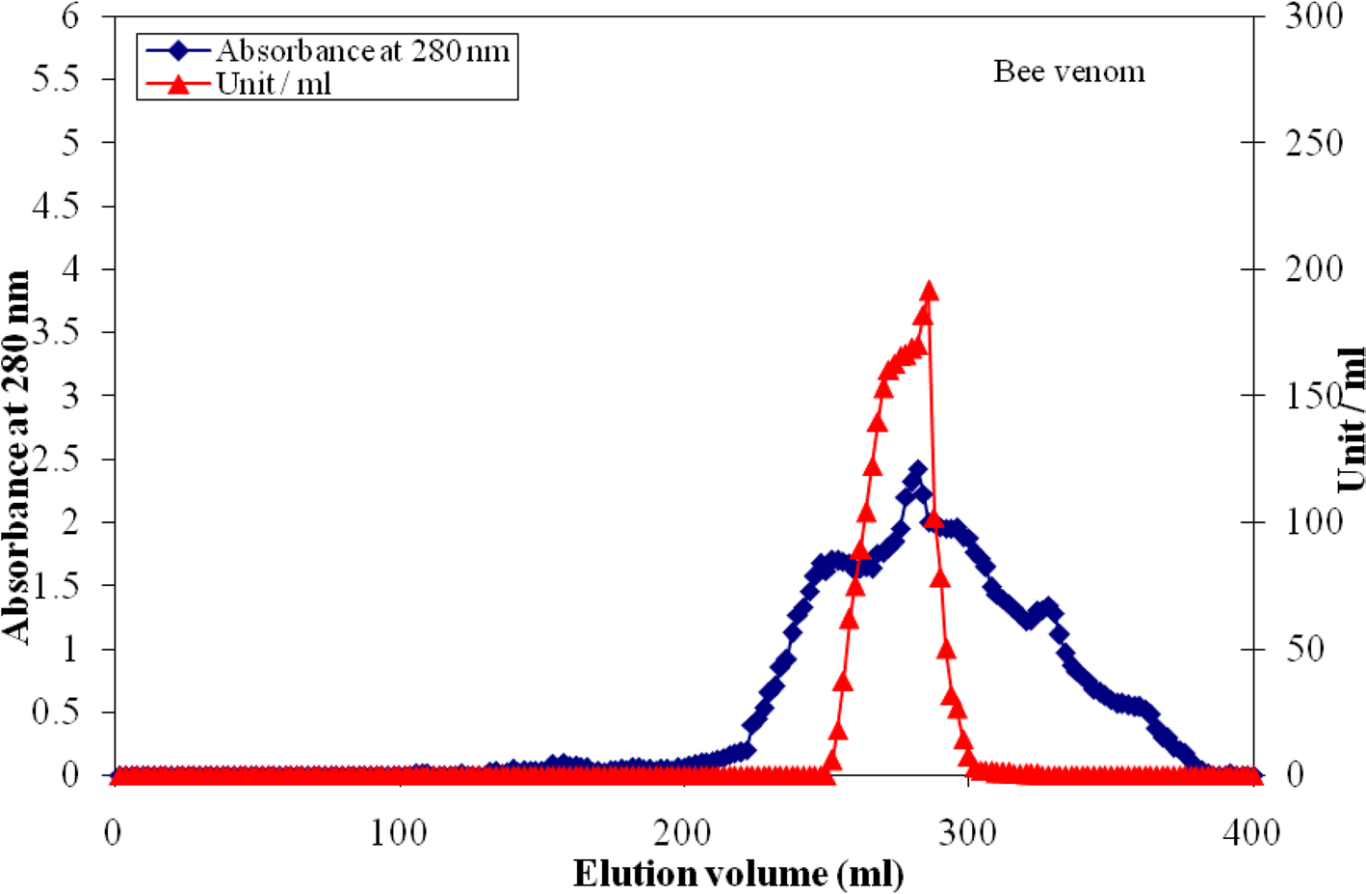
A chromatographic typical elution profile for the concentrated pooled DEAE-cellulose fractions containing superoxide dismutase enzyme activity on Sephacryl S-300 column (142 cm × 1.75 cm i.d.) previously equilibrated with 20 mmol L.^−1^ potassium phosphate buffer (pH 7.4)

The superoxide dismutase starting specific activity in venom extract was 321 units/mg protein. One peak of superoxide dismutase activity (BVSOD) was resolved from DEAE cellulose and was eluted with 0 M NaCl of 20 mmol L^−1^ potassium phosphate buffer (pH 7.4) (Fig. 1). BVSOD-specific activity of DEAE-cellulose pooled fraction was increased 1.83-fold over venom crude extract with 53.9% recovery. The elution profile of Sephacryl S-300 column showed one peak of superoxide dismutase activity (Fig. 2). After chromatography, the specific activity of superoxide dismutase enzyme was raised to 1250 units/mg protein that represent 3.89-fold with 21.0% yield (Table 1). The molecular weight of BVSOD was determined from gel filtration column elution volume to be 32 kDa.

**Table 1:** A Typical purification scheme of BVSOD

Purification steps	Total protein (mg)	Total activity (unit)	Specific activity	Yield (%)	Fold purification
Crude honey Bee venom	68.9	22,128	321	100	1.00
DEAE cellulose					
SOD (0 M NaCI)	20.3	11,949	588	53.9	1.83
Sephacryl S-300					
SOD	3.72	4653	1250	21.0	3.89

### Electrophoretic analysis

**Fig. 3:**
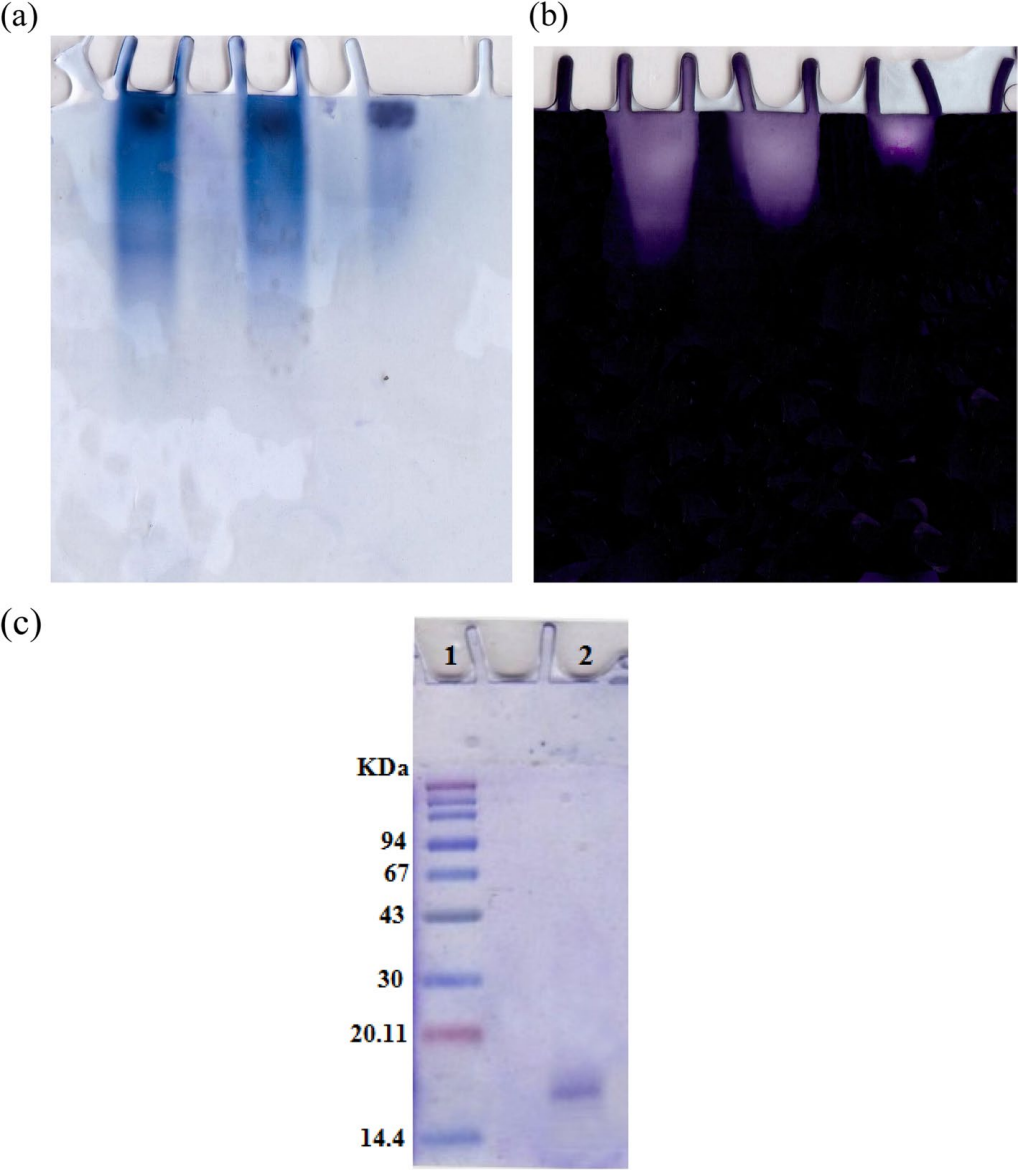
**a** Electrophoretic analysis of superoxide dismutase (enzyme protein pattern) of the different purification steps on 7% native polyacrylamide gel: (1) crude venom, (2) DEAE-cellulose fraction, and (3) Sephacryl S-300 purified fraction of BVSOD. **b** Electrophoretic analysis of superoxide dismutase (enzyme activity pattern) of the different purification steps on 7% native polyacrylamide gel: (1) crude venom, (2) DEAE-cellulose fraction, and (3) Sephacryl S-300 purified fraction of BVSOD. **c** Subunit molecular weight determination by electrophoretic analysis of purified BVSOD on 12% SDS–polyacrylamide gel: (1) molecular weight marker proteins and (2) purified superoxide dismutase

Purification steps, venom extract, DEAE cellulose, and Sephacryl S-300 fraction were loaded on 7% native PAGE electrophoresis. One band of protein agreed with SOD activity band denoting purity of BVSOD preparation (Fig. 3 a and b). Electrophoretic analysis of BVSOD on SDS/PAGE was compared to markers protein and showed its molecular weight subunit to be 16 kDa (Fig. 3c).

### Effect of pH

**Fig. 4:**
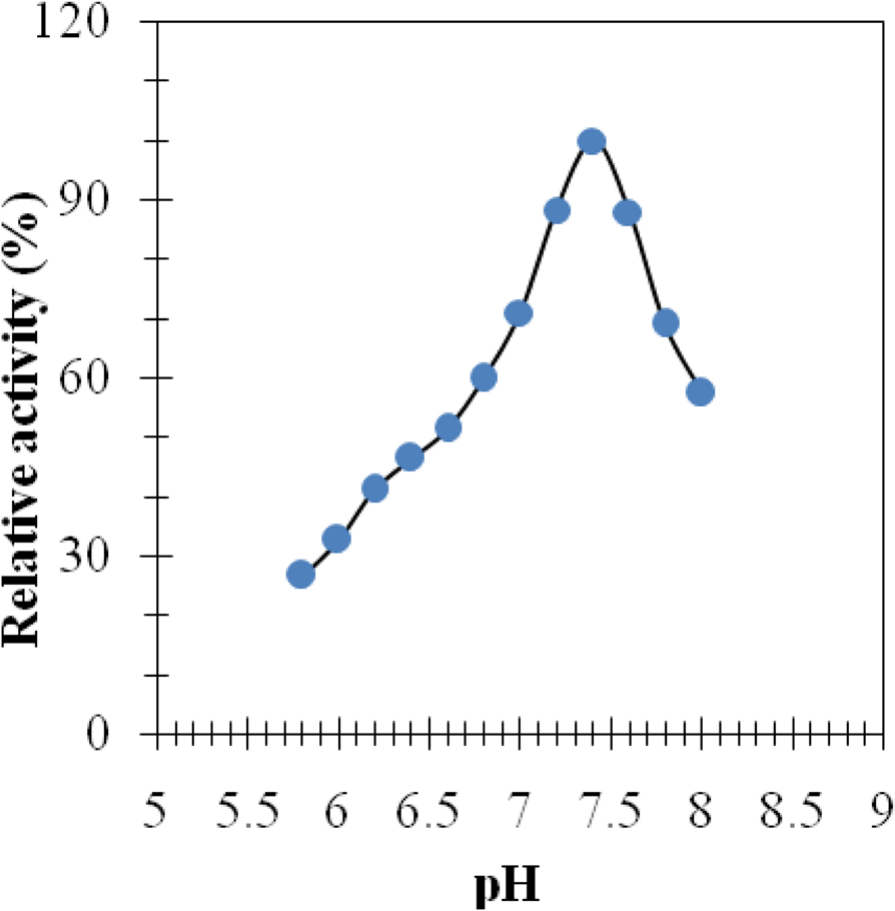
pH effect on purified BVSOD using 0.02 M phosphate buffer, pH (5.8–8.0)

The pH effect on purified BVSOD was carried out utilizing buffer 20 mmol L^−1^ potassium phosphate with values of pH between (5.7 and 8.0). The highest activity of BVSOD was recorded at pH 7.4 (Fig. 4).

### Effect of cations and inhibitors

The divalent cations effect and inhibitors effect on activity of purified BVSOD was measured after preincubation at 37 °C with 2 and 5 mM of each cation and each inhibitor. A control without cations and inhibitors was taken 100% activity. CuCl_2_, ZnCl_2_, and MgCl_2_ elevated activity of BVSOD, while FeCl_2_, CoCl_2_, and NiCl_2_ inhibited activity of BVSOD (Table 2). Potassium cyanide and hydrogen peroxide were most potent inhibitors for BVSOD activity. DL-Dithiothreitol, EDTA, 1,10-phenanthroline, *β*-mercaptoethanol, iodoacetamide, phenylmethylsulfonyl fluoride (PMSF), and potassium dichromate inhibited BVSOD activity (Table 3).

**Table 2:** Effect of divalent cations on BVSOD

Reagent	Final concentration (mM)	Residual activity (%)
Control	––-	100.0
CaCl_2_	2.0	99.4
	5.0	99.1
CoCl_2_	2.0	80.6
	5.0	59.7
CuCl_2_	2.0	116
	5.0	128
FeCl_2_	2.0	44.1
	5.0	15.9
MgCl_2_	2.0	113
	5.0	121
MnCl_2_	2.0	101
	5.0	99.8
NiCl_2_	2.0	73.4
	5.0	31.5
ZnCl_2_	2.0	118
	5.0	131

**Table 3:** Effect of various inhibitors on BVSOD

Reagent	Final concentration (mM)	Inhibition (%)
Control	––-	0.0
Potassium cyanide (KCN)	2.0	83.6
	5.0	98.3
Hydrogen peroxide (H_2_O_2_)	2.0	79.4
	5.0	95.1
Sodium azide (NaN_3_)	2.0	26.8
	5.0	37.6
Sodium dodecyl sulfate (SDS)	2.0	10.6
	5.0	13.3
Ethylenediaminetetraacetic acid (EDTA)	2.0	43.1
	5.0	51.7
DL-Dithiothreitol (DTT)	2.0	28.3
	5.0	36.7
β-Mercaptoethanol	2.0	34.5
	5.0	41.8
1,10-Phenanthroline	2.0	20.4
	5.0	28.6
Phenylmethylsulfonyl fluoride (PMSF)	2.0	17.3
	5.0	19.6
Iodoacetamide	2.0	30.4
	5.0	39.2
Potassium dichromate (K_2_Cr_2_O_7_)	2.0	52.8
	5.0	71.3

### Antimicrobial and antitumor activity

**Fig. 5:**
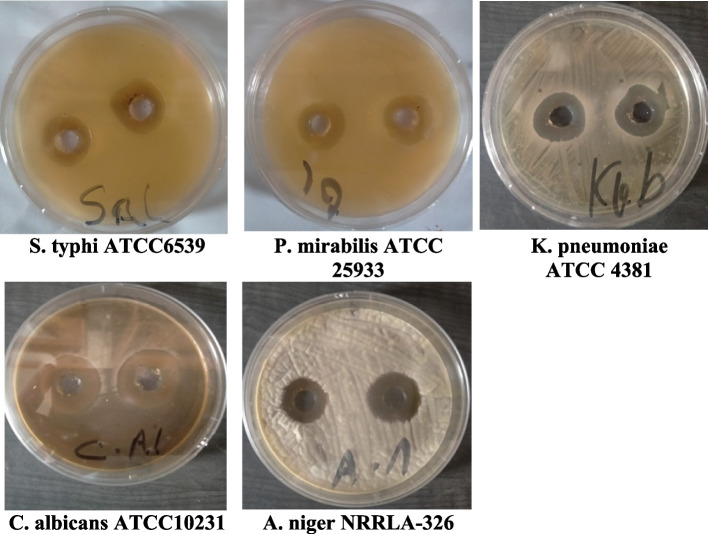
Antimicrobial activity of purified BVSOD

**Fig. 6:**
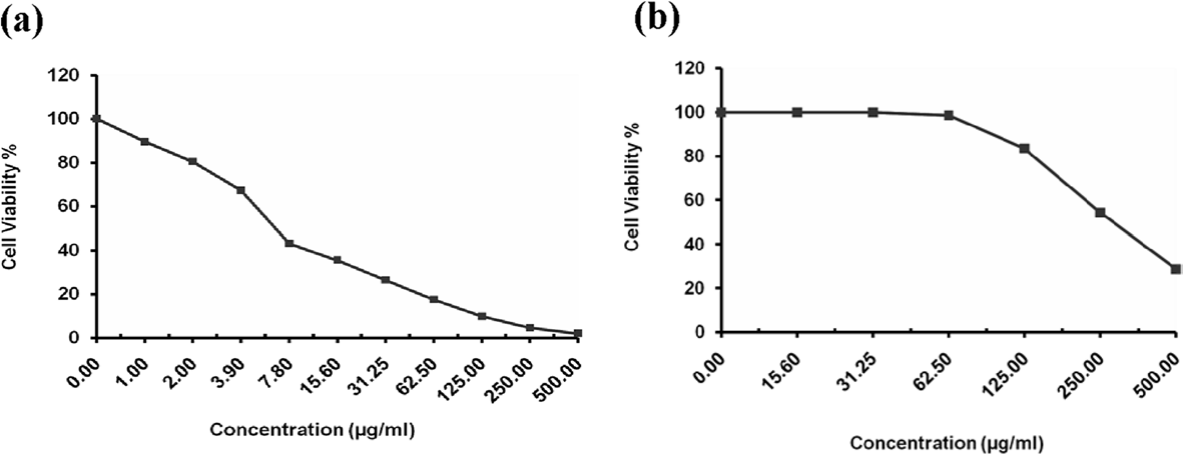
**a** Inhibitory activity of purified BVSOD against hepatocellular carcinoma cells. **b** Inhibitory activity of purified BVSOD against breast carcinoma cells

**Fig. 7:**
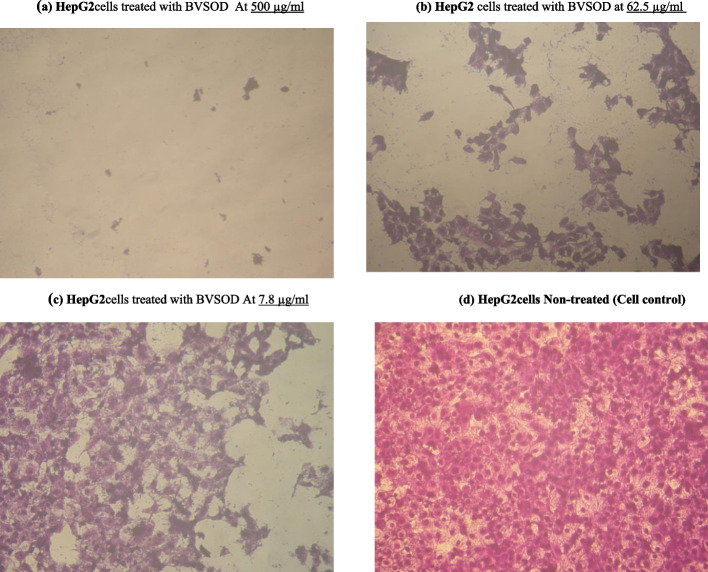
Morphological evaluation of cytotoxicity of purified BVSOD against HepG2 cell line using different concentrations and compared with HepG2 non-treated cell as a control. **a** HepG2 cells treated with BVSOD at 500 µg/ml. **b** HepG2 cells treated with BVSOD at 62.5 µg/ml. **c** HepG2 cells treated with BVSOD at 7.8 µg/ml. d HepG2cells non-treated (cell control)

The purified BVSOD was screened for its antimicrobial activity. All data expressed as the mean of three reads, while no difference between reads has been observed. The inhibition zone for purified BVSOD was determined and compared with the reference standard (Fig. 5). Results of antimicrobial screening are shown in Table 4. The data obtained showed that BVSOD have an antimicrobial activity. Results showed that the BVSOD exhibited good antibacterial activity against *Proteus mirabilis* ATCC 25,933, *Salmonella typhi* ATCC6539, and *Klebsiella pneumoniae* ATCC 43,816 with inhibition zone (13 mm, 15 mm, and 15 mm), respectively. On the other hand, the antifungal activity of BVSOD was also measured, and the obtained results showed that BVSOD displayed a potent anticandidal activity with inhibition zone 22 mm, while the activity was also strong against *Aspergillus niger* with inhibition zone 18 mm. The purified BVSOD was screened for its antitumor activity (Tables 5) using human hepatocellular carcinoma cell line (HepG-2 cells) and human breast cancer cell line (MCF-7 cells). BVSOD was found to have antitumor activity (Figs. 6 and 7).

**Table 4:** Determination of antimicrobial activity

Sample	*P. mirabilis* ATCC 25,933	*K. pneumoniae* ATCC 43,816	*S. typhi* ATCC6539	*C. albicans* ATCC10231	*A. niger* NRRLA-326
Oxytetracycline	25.0 ± 0.00 mm	10.0 ± 0.00 mm	10.0 ± 0.00 mm	− ve	7.0 ± 0.00 mm
BVSOD	13.0 ± 0.00 mm	15.0 ± 0.00 mm	15.0 ± 0.00 mm	22.0 ± 0.00 mm	18.0 ± 0.00 mm

All data expressed as the mean of three reads, while no difference between reads has been observed. Oxytetracycline (OT) 30 µg was used as reference standard

**Table 5:** Determination of antitumor activity

Sample conc. (µg/ml)	Viability %	Inhibitory %	SD ( ±)
(a) Inhibitory activity against hepatocellularcarcinoma cells was under these experimental conditions with *IC*_50_ = 6.67 ± 0.75 µg/ml			
500	1.97	98.03	0.31
250	4.52	95.48	0.26
125	9.73	90.27	0.95
62.5	17.40	82.6	1.42
31.25	26.35	73.65	1.73
15.6	35.42	64.58	1.64
7.8	42.97	57.03	2.59
3.9	67.21	32.79	2.37
2	80.36	19.64	1.08
1	89.42	10.58	0.84
0	100	0	0
(b) Inhibitory activity against breast carcinoma cells was detected under these experimental conditions with *IC*_50_ = 291.1 ± 9.54 µg/ml			
500	28.63	71.37	3.95
250	54.21	45.79	2.43
125	83.59	16.41	1.73
62.5	98.64	1.36	0.82
31.25	100	0	0
15.6	100	0	0
0	100	0	0

## Discussion

It is well established that SOD (EC 1.15.1.1) serves a key antioxidant role [35]. SODs have been studied in venoms of many parasitoids such as *Cotesia chilonis*, *Leptopilina boulardi*, *Diversinervus elegans*, and *Tetrastichus brontispae* [36–38]; these studies suggest that SOD is present in parasitoids venom. The present study demonstrated a simple and reproducible purification method of the superoxide dismutase from venom of Egyptian honeybee *Apis mellifera lamarckii*. The BVSOD was isolated from *Apis mellifera lamarckii* by two chromatographic steps. The chromatographic profile obtained from DEAE-cellulose column showed a well-defined fraction identified as BVSOD. The pooled fraction-specific activity of BVSOD was increased 1.83-fold over venom extract with 53.9% recovery. After chromatography on Sephacryl S-300 column, BVSOD-specific activity was raised to 1250 units/mg protein that represent purification fold of 3.89 and 21.0% yield. Present findings are comparative to SOD of Italian honeybee *Apis mellifera* royal jelly previously isolated with 53.05 units/mg specific activity [39]. Purification steps are investigated by 7% native PAGE electrophoresis. One protein band agreed with BVSOD activity band denoting purity of BVSOD preparation. The molecular weight of BVSOD was investigated from gel filtration column elution volume to be 32 kDa. Electrophoretic analysis of BVSOD and protein markers on SDS/PAGE was compared and showed BVSOD molecular weight subunit to be 16 kDa; this is consistent with SOD previously isolated from silkworm, *Bombyx mori* [40], with SOD isolated from *Drosophila melanogaster* [41] and 29.3 kDa from midgut of *Helicoverpa armigera* larvae [42]. The effect of pH on the purified BVSOD was carried out utilizing buffer 20 mmol L^−1^ potassium phosphate with values of pH between 5.7 and 8.0. The highest activity of BVSOD was recorded at pH 7.4, while midgut of *Helicoverpa armigera* larvae SOD showed maximum activity at pH 11.0 [42]. CuCl_2_, ZnCl_2_, and MgCl_2_ elevated the activity of BVSOD, while FeCl_2_, CoCl_2_, and NiCl_2_ inhibited activity of BVSOD. These results are in accordance with SOD from muscle tissue of the shrimp *Macrobrachium nipponense* and from *Marinomonas* sp. bacteria which were markedly enhanced by ZnCl_2_ and inhibited by FeCl_2_ [43, 44]. Differentiation between SODs classes is based on selective chemicals inhibition [45, 46]. Types of SODs can be differentiated by its inhibition susceptibility to hydrogen peroxide (H_2_O_2_) and potassium cyanide (KCN) [47–49]. It is well-known that the superoxide dismutase isoenzyme that is very sensitive to potassium cyanide inhibition and inhibited with hydrogen peroxide is cupper/zinc containing isoenzyme [10, 50]. In this study, potassium cyanide (KCN) and hydrogen peroxide (H_2_O_2_) are found to be potent inhibitors of purified BVSOD activity suggesting that it is a copper/zinc containing enzyme. 1,10-Phenanthroline and EDTA-inhibited BVSOD activity indicate that BVSOD is a metalloenzyme. Dithiothreitol and *β*-mercaptoethanol-inhibited BVSOD activity indicate that active site containing − SH groups play a major role for enzyme activity. Similarly, SOD of *Radix lithospermi* seed is sensitive to thiol compounds [46]. PMSF and iodoacetamide inhibited BVSOD activity indicating that serine, cysteine, methionine, and histidine residues have important effects on enzyme structure and activity; these results are in accordance with SOD of chicken liver [47]. The activity inhibition of BVSOD with K_2_Cr_2_O_7_ may be due to metal prosthetic groups oxidation which is a principle for enzyme activity. SOD of muscle tissue of the shrimp is also susceptible to K_2_Cr_2_O_7_ [43]. Antimicrobial activity results showed that BVSOD have a good antibacterial activity against *Proteus mirabilis* ATCC 25,933, *Salmonella typhi* ATCC6539, and *Klebsiella pneumoniae* ATCC 43,816. BVSOD displayed a potent anticandidal activity, while the activity was also strong against *Aspergillus niger*. BVSOD was screened for its antitumor activity using human hepatocellular carcinoma cell line (HepG-2 cells) and human breast cancer cell line (MCF-7 cells). BVSOD was found to have a potent antitumor activity against HepG-2 cells and have an inhibitory effect against MCF-7 cells. A total of 500 µg of BVSOD causes 98.03 inhibition for HepG-2 cells, and 1 µg of BVSOD causes 10.58 inhibition. The 50% inhibitory concentration (*IC*_50_) of BVSOD against HepG2 cell was estimated to be 6.67 µg. Morphological evaluation of cytotoxicity of BVSOD against HepG2 cell line was carried out using different concentrations of BVSOD and compared with HepG2 non-treated cell as a control.

## Conclusions

This study presents a simple, fast, and reproducible isolation and characterized protocols of SOD from venom of Egyptian honeybee *Apis mellifera lamarckii*. The method can scaled up from the laboratory level to semi-pilot and pilot levels for production of this enzyme in larger scales. The purified BVSOD found to have antimicrobial and antitumor activities which can be used for various medical and clinical applications. The present study will help in finding the optimum conditions for the enzyme activity, and this will be helpful in BVSOD uses in various applications with high efficiency.

## Data Availability

All data generated or analyzed during this study are included in this published article.

## Abbreviations

BVSOD: Bee venom superoxide dismutase
NBT: Nitroblue tetrazolium
DMSO: Dimethyl sulfoxide
PMS: Phenyl methosulfate
PMSF: Phenylmethylsulfonyl fluoride
DTT: 1,4-Dithiothreitol
BSA: Bovine serum albumin
DEAE-cellulose: Diethylaminoethyl cellulose
DMEM: Dulbecco’s Modified Eagle Medium
HEPES: (4-(2-Hydroxyethyl)-1-piperazineethanesulfonic acid)
HepG-2 cells: Human hepatocellular carcinoma cell line
MCF-7 cells: Human breast cancer cell line
